# Innate Immune Response of Primary Human Keratinocytes to West Nile Virus Infection and Its Modulation by Mosquito Saliva

**DOI:** 10.3389/fcimb.2018.00387

**Published:** 2018-11-02

**Authors:** Magali Garcia, Haoues Alout, Fodé Diop, Alexia Damour, Michèle Bengue, Mylène Weill, Dorothée Missé, Nicolas Lévêque, Charles Bodet

**Affiliations:** ^1^Laboratoire de Virologie et Mycobactériologie, CHU de Poitiers, Poitiers, France; ^2^Laboratoire Inflammation, Tissus Epithéliaux et Cytokines, EA 4331, Université de Poitiers, Poitiers, France; ^3^Institut des Sciences de l'Evolution, Université de Montpellier, Montpellier, France; ^4^MIVEGEC UMR 224, Université de Montpellier, IRD, CNRS, Montpellier, France

**Keywords:** West Nile virus, keratinocytes, mosquito, saliva, immunomodulation, innate immune response, viral replication, interferon-stimulated genes

## Abstract

West Nile Virus (WNV) is a flavivirus involved in many human infections worldwide. This arthropod-borne virus is directly co-inoculated with mosquito saliva through the epidermis and the dermis during blood meal. WNV starts replicating in the skin before migrating to the draining lymph node, leading to widespread viremia and in some cases to neurological symptoms. Skin is a complex organ composed of different cell types that together perform essential functions such as pathogen sensing, barrier maintenance and immunity. Keratinocytes, which represent 90% of the cells of the epidermis, are the organism's first line of defense, initiating innate immune response by recognizing pathogens through their pattern recognition receptors. Although WNV was previously known to replicate in human primary keratinocytes, the induced inflammatory response remains unknown. The aim of this study was first to characterize the inflammatory response of human primary keratinocytes to WNV infection and then, to assess the potential role of co-inoculated mosquito saliva on the keratinocyte immune response and viral replication. A type I and III interferon inflammatory response associated with an increase of IRF7 but not IRF3 mRNA expression, and dependent on infectious dose, was observed during keratinocyte infection with WNV. Expression of several interferon-stimulated gene mRNA was also increased at 24 h post-infection (p.i.); they included CXCL10 and interferon-induced proteins with tetratricopeptide repeats (IFIT)-2 sustained up until 48 h p.i. Moreover, WNV infection of keratinocyte resulted in a significant increase of pro-inflammatory cytokines (TNFα, IL-6) and various chemokines (CXCL1, CXCL2, CXCL8 and CCL20) expression. The addition of *Aedes aegypti* or *Culex quinquefasciatus* mosquito saliva, two vectors of WNV infection, to infected keratinocytes led to a decrease of inflammatory response at 24 h p.i. However, only *Ae. Aegypti* saliva adjunction induced modulation of viral replication. In conclusion, this work describes for the first time the inflammatory response of human primary keratinocytes to WNV infection and its modulation in presence of vector mosquito saliva. The effects of mosquito saliva assessed in this work could be involved in the early steps of WNV replication in skin promoting viral spread through the body.

## Introduction

Since its discovery in 1937 from a febrile woman in Uganda, West Nile virus (WNV) has been involved in mild febrile disease outbreaks in Africa, Asia and Europe (Lim et al., 2011). It is now endemic on the North American continent after its introduction in 1999 (Lanciotti et al., 1999) and propagating southward. WNV is considered the first cause of viral encephalitis worldwide (Chancey et al., 2015; David and Abraham, 2016). In about 20% of human cases of WNV infection, a mild febrile disease appears after 2–14 days of incubation (Lindsey et al., 2012; Sejvar, 2016). Less than 1% of infected patients then develop a WNV neuroinvasive disease such as aseptic meningitis, encephalitis, or acute poliomyelitis-like syndrome that can be life-threatening (Campbell et al., 2002).

WNV is an arthropod-borne virus belonging to the *Flavivirus* genus, as do Dengue (DENV), Zika (ZIKV) or yellow fever viruses. WNV primary hosts are birds while mammals, particularly humans and horses, represent accidental and dead-end hosts infected through the inoculation by infected female mosquitoes (Kramer et al., 2008). *Culex* mosquitoes participate in an enzootic cycle between birds and represent a “bridge” vectors between the avian and mammalian hosts (Turell et al., 2005) because of their opportunistic feeding behavior.

Virus inoculation in the skin is a key step in the pathophysiology of WNV infection. Thereby, skin constitutes not only the first site of viral replication in host but also the initiation site of the antiviral immune response. Skin is organized in three successive layers, the epidermis, the most superficial, the dermis and the hypodermis, the deepest. The multi-layered epidermis consists mainly of keratinocytes that express pathogen recognition receptors (PRRs) involved in the recognition of highly conserved pathogen molecular patterns (PAMPs) among microorganisms (Lebre et al., 2007). Once activated by a PAMP, transmembranar toll-like receptors (TLRs) and/or retinoic acid-inducible gene (RIG)-I-like receptors (RLRs) such as RIG-I and melanoma differentiation antigen 5 (MDA5) trigger downstream signaling pathways contributing to the induction of an innate immune response (Akira, 2006). During blood-feeding, WNV is co-inoculated with mosquito saliva predominantly in the extravascular space of the skin (Styer et al., 2007) and starts to replicate in keratinocytes (Lim et al., 2011). Mosquito saliva contains many proteins that modulate host hemostasis and immune response, facilitating blood feeding but also virus transmission (Ribeiro, 2000; Schneider and Higgs, 2008; Styer et al., 2011; Moser et al., 2016).

WNV is a positive-sense, single-stranded (ss) RNA virus transcribed in a complementary negative RNA, thereby constituting a double-stranded replicative form (ds-RF) (Brinton, 2013). The negative ssRNA is used in turn as a template for simultaneous synthesis of multiple positive ssRNA constituting partial double-stranded (ds) replicative intermediates (RI) (Brinton, 2013).

Thus, once injected in the skin and starting replicating, flaviviral RNAs are recognized as PAMPs in the cells either as ssRNA sensed by TLR7 and RIG-I or dsRNA recognized by TLR3, RIG-I and MDA5 (Westaway et al., 1999; Akira, 2006; Shipley et al., 2012). PRR activation by flaviviral PAMPs leads to expression of chemokines, cytokines, type I and type III interferons (IFNs) and interferon-stimulated genes (ISGs) by skin cells (Garcia et al., 2017). Nonetheless, although innate immune response of cutaneous cells to DENV or ZIKV has been investigated (Surasombatpattana et al., 2011; Hamel et al., 2015), the inflammatory response of WNV infected keratinocytes remains unknown. The aim of this study was to characterize the antiviral response of human primary keratinocytes during WNV infection. Moreover, as mosquito saliva can exert wide effects promoting blood-meal and infection, we investigated the effects of the saliva of two mosquito species on WNV replication and inflammatory response induced in these cells.

## Material and methods

### Isolation and culture of normal human epidermal keratinocytes from skin samples

The Ethics Committee of the Poitiers Hospital approved the use of human skin samples for research studies. All subjects gave written informed consent in accordance with the Declaration of Helsinki. Normal abdominal or breast skin was obtained from patients undergoing plastic surgery. Small pieces of skin were thoroughly washed with phosphate-buffered saline solution free of calcium and magnesium (PBS; Gibco) after removal of fat. The skin was minced into fragments of about 125 mm^2^ using scalpel blades. Skin samples were incubated overnight at 4°C in a dispase solution (25 U/mL; Life Technologies). Epidermal sheets were removed from the dermis, and keratinocytes were dissociated by trypsin digestion (trypsin-EDTA; Gibco) for 15 min at 37°C. The cell suspension was then filtered through a 280 μm sterile filter. Dulbecco's modified essential medium (DMEM; Gibco) supplemented with 10% of fetal bovine serum (FBS; Gibco) was added vol/vol and the suspension was centrifuged at 300 × g for 10 min. Keratinocytes were seeded at a density of 10^7^ cells in 75-cm^2^ tissue culture flask in Keratinocyte-Serum Free Medium (K-SFM) supplemented with bovine pituitary extract (25 μg/mL) and recombinant epidermal growth factor (EGF) (0.25 ng/mL; all were purchased from Invitrogen, Life Technologies). The cultures were incubated at 37°C in a humidified atmosphere with 5% CO_2_ until confluence and then stored frozen in liquid nitrogen until use. Finally, keratinocytes were seeded in sterile 24-well culture plates at a density of 4 × 10^4^ cells/well in K-SFM supplemented with bovine pituitary extract and EGF and cultured to 80% confluence. Cells were then starved overnight in K-SFM alone before stimulation.

### WNV strain production

A lineage 1 clinical strain of WNV was used in this study. The strain isolated from a human brain during the epidemic that occurred in Tunisia in 1997 was provided by Dr I. Leparc Goffart (French National Reference Center on Arboviruses, Marseille, France). The viral stock was produced on the *Ae. albopictus* clone C6/36 cells (ATCC® CRL-1660™). Cells were cultivated in Leibovitz's L-15 medium (Gibco) supplemented with 2 % of tryptose-phosphate (Gibco) and 5 % of FBS in 75-cm^2^ tissue culture flask at 28°C until 50% of confluency and then infected at a Multiplicity Of Infection (MOI) of 0.01 for 72 h. Cell supernatant of infected cells and uninfected C6/36 cell used for control, were clarified by centrifugation in 50 ml tubes for 15 min at 1,500 × g. Then, the viral suspension and the supernatant from uninfected C6/36 suspension were ultrafiltrated in amicon ultra-4 centrifugal filter units 100 kD (Dutscher) for 5 min at 3,000 × g. The viral suspension and the supernatant from uninfected C6/36 suspension were finally frozen at −80°C in cryotubes containing 500 μL of Leibovitz's L-15 medium supplemented with 0.5 M sucrose and 50 mM HEPES. The final viral titer was 10^7.97^ TCID_50_ (Tissue Culture Infection Dose) per mL as determined by plaque assays on Vero cell monolayers.

### Mosquito saliva

Saliva were obtained from 7-day-old adult females *Aedes aegypti* (Bora-Bora strain) and *Culex quinquefasciatus* (Slab strain) mosquitoes grown in insectary at 27°C under 70 ± 8% relative humidity and 12:12 light and dark photoperiod. On the hatching day, larvae were equally seeded into plastic trays containing water. Larvae were fed *ad libitum* with a mixture of rabbit and fish-food whilst adults were fed with 10% sucrose solution [w/v]. Salivation was performed on individual mosquitoes according to an adapted protocol from (Dubrulle et al., 2009). Each individual was chilled to remove legs and wings. The mosquito's proboscis was then inserted into a micropipette tip containing 10 μL of DMEM with protease inhibitor (Gibco). After 30 min, the saliva-containing DMEM was expelled and collected into an 1.5 ml tube.

### Viral infection

Human primary keratinocyte cultures (60–80% of confluency) from 6 to 8 different patients were infected at MOI of 0.1, 1, and 10 and incubated for 24, 48, and 72 h at 37°C in 5% CO_2_ in K-SFM medium. Mocked-infected keratinocytes incubated with uninfected C6/36 cell supernatant were used as control. For experiments assessing the role of saliva, keratinocytes were infected as previously described in presence of 0.5 μg/L of mosquito saliva for 24 or 48 h.

Cell culture supernatants and cell monolayers were collected at each point of the time course infection in order to perform viral quantification by RT-qPCR and transcriptomic analysis of inflammatory marker expression as described below.

### RNA extraction, reverse transcription and real-time PCR analysis

#### RNA extraction

For viral RNA quantification in cell supernatant, Total DNA/RNA of 200 μL of keratinocyte supernatant was extracted on NucliSENS easyMAG® automated system (bioMérieux) according to manufacturer's recommendations. For intracellular viral RNA quantification and evaluation of the host inflammatory response, total RNA extraction from keratinocyte monolayer was performed using the Nucleo-Spin XS RNA extraction kit according to the manufacturer's instructions (Macherey-Nagel). RNA concentrations and purity were determined using the Nanodrop 2000 spectrophotometer (Thermo Fisher Scientific).

#### Viral quantification by RT-qPCR

Viral quantification in cell supernatants and keratinocytes was performed using a one-step real time RT-PCR assay from 5 μL of total RNA in 96-well plates on Applied Biosystems 7500 thermocyler. Reaction mixtures consisted of 12.5 μL of Master Mix (Invitrogen), 0.5 μL (0.2 μM) of forward (5′-3′ GTGCGGTCTACGATCAGTTT) and reverse primers (5′-3′ CACTAAGGTCCACACCATTCTC), and 0.25 μL (0.1 μM) of 5′FAM and 3′Dark Quencher probe (5′-3′ AATGTGGGAAGCAGTGAAGGACGA), 0.5 μL of SuperScript III reverse transcriptase (Invitrogen) and DNA polymerase platinium Taq (Invitrogen), 0.5 μL of RNase out (Invitrogen) and 5.25 μL of water. The calibration range was performed using a transcript produced using a plasmid containing WNV genome deleted from genes coding structural proteins provided by Dr P.W. Mason (Microbiology and Immunology department, Texas University, Galveston, USA). The transcripts were diluted in order to get a calibration range allowing the quantification of viral load from 10^2^ to 10^7^ RNA copies/mL. Each standard of the calibration curve was added in duplicate to each analysis.

#### Transcriptomic analysis of the innate antiviral immune response in keratinocytes

Total RNA (1 μg) was reverse transcribed using SuperScript II kit (Invitrogen). Quantitative real time PCR was performed in 96-well plates using LightCycler-FastStart DNA Master^Plus^SYBR GREEN I kit (Roche) on LightCycler 480 (Roche). Reaction mixtures consisted of 1X DNA Master Mix, 1 μM forward and reverse primers designed using Primer 3 software and 12.5 ng of cDNA template in a total volume of 10 μl. PCR conditions were as follows: 5 min at 95°C, 40 amplification cycles comprising 20 s at 95°C, 15 s at 64°C, and 20 s at 72°C. Samples were normalized with regard to two independent control housekeeping genes (Glyceraldehyde-Phospho-Dehydrogenase and 28S rRNA gene) and reported according to the ΔΔCT method as RNA fold increase: 2^ΔΔ^ CT = 2^ΔCTsample−ΔCTreference^.

### Viral quantification by end-point dilution assay

Vero cells were seeded, in 96-well plates, the day before titration at the rate of 4 × 10^3^ cells/well in DMEM (Gibco) supplemented with 2% SVF. The suspension was successively diluted from 10^−1^ to 10^−9^ in DMEM medium supplemented with 2% SVF. Then, 100 μL of each dilution were deposited in a row of 6 wells. A reading was performed after 96 h of incubation at 37°C in an atmosphere containing 5% CO_2_. The wells in which the cells had a cytopathic effect were considered positive for viral infection. The titer of the viral suspension was then determined using the Kärber's method for assessing the 50% tissue culture infective dose (TCID50).

### Enzyme-linked immunosorbant assay (ELISA)

Cell culture supernatants were UV inactivated at 2 joules/cm^2^ for 10 min using a Bio-Link crosslinker in order to inactivate virus. Levels of IFNβ and CCL20 in cell culture supernatants were determined for each sample using human ELISA kits (R&D systems for IFNβ; PeproTech for CXCL10) in accordance with the manufacturers' specifications.

### Statistical analysis

Results were analyzed by GraphPad Prism version 5. The statistical significance of the difference between two groups was evaluated by the Wilcoxon's test. Differences were considered to be significant at *p* < 0.05.

## Results

### Primary human keratinocytes are permissive to WNV infection

In a first step, the capacity of WNV to replicate in epidermal keratinocytes from different patients was evaluated at MOI of 0.1, 1, and 10.

**Figure 1:**
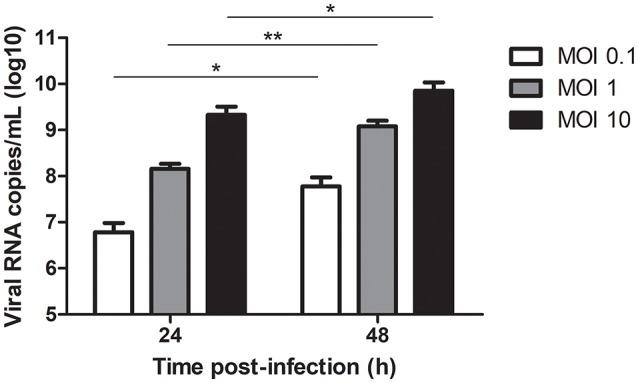
WNV amplification in RNA copies/ml in 24 and 48 h-infected keratinocyte supernatants at MOI of 0.1, 1, and 10. Data are represented as mean + SEM of 6–8 independent experiments. ^*^p < 0.05 and ^**^p < 0.01.

The viral quantification of WNV in keratinocyte supernatant using RT-PCR analysis showed an increase of WNV viral load of about 1 log per 24 h during the 48 first hours of keratinocyte infection for all the MOI tested (Figure 1). At 72 h p.i., WNV RNA production increased by 2-fold only for the MOI of 0.1 and 1, the MOI of 10 resulting in massive lysis of keratinocytes (data not shown).

### Interferon and interferon stimulated-gene mRNA expression in keratinocytes infected by WNV

**Figure 2:**
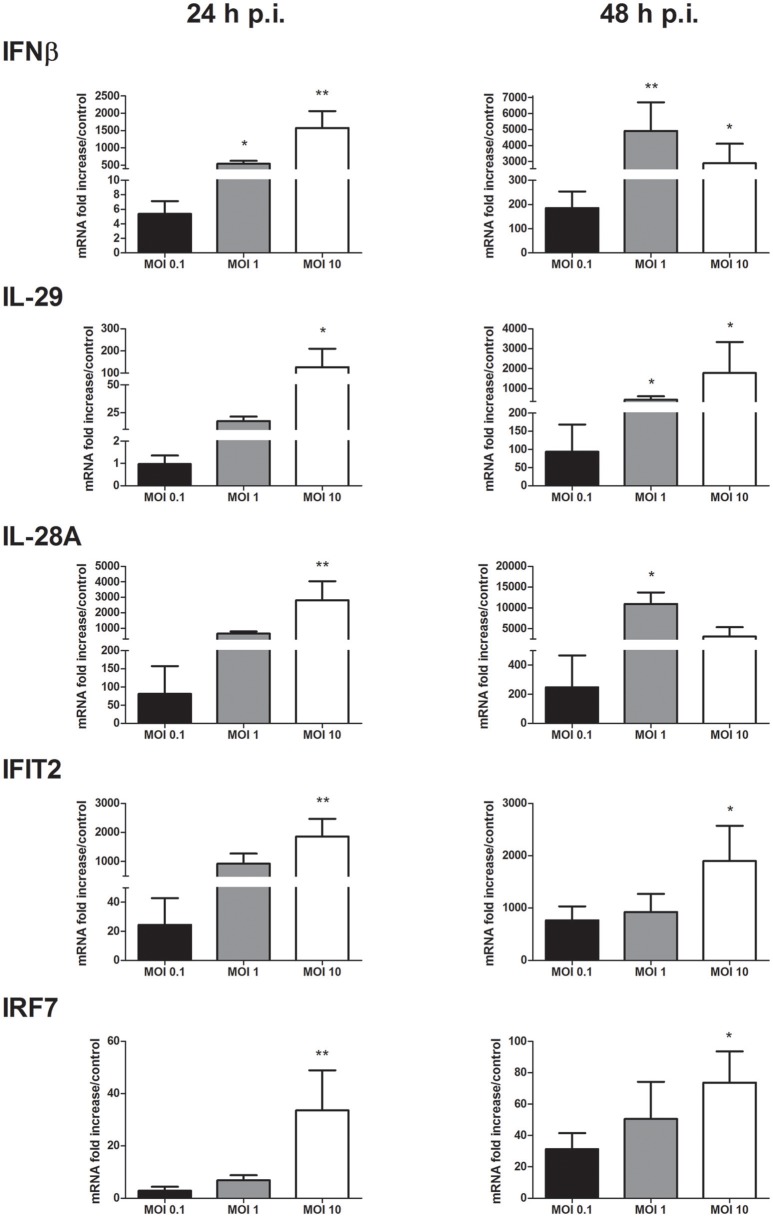
IFN and ISG mRNA expression in response to keratinocyte infection by WNV. IFNβ, IL-29, IL-28A, IFIT2, and IRF7 mRNA expression in human primary keratinocytes infected with WNV for 24 and 48 h, at MOI of 0.1, 1, and 10. mRNA expression levels are expressed as the fold increase above mock-infected cultures. Data are represented as mean + SEM of four independent experiments. ^*^p < 0.05 and ^**^p < 0.01.

The IFN antiviral response was then characterized in keratinocytes infected with WNV at MOI of 0.1, 1, and 10. A significant induction of type I and III IFN expression such as IFNβ, IFNλ1 (interleukin (IL)-29) and λ2 (IL-28A) was observed as soon as 24 h p.i. and sustained at 48 h p.i. in a MOI-dependent manner (Figure 2). IFNβ and IL-28A mRNA expression was induced at 24 h p.i. from 5 to 1,570 times and from 80 to 2,800 times, respectively. In a consistent way, ISG such as IFIT1 (data not shown), IFIT2, IFIT3 (data not shown) and IRF7 mRNA expression was induced (Figure 2). IFIT2 mRNA expression was increased from 20 to 1,860 times at 24 h p.i. at MOI of 0.1 and 10, respectively. On the other side, IRF3 mRNA levels were not modulated in comparison to mock-infected keratinocytes (data not shown).

### Cytokine and chemokine mRNA expression in response to keratinocyte infection with WNV

**Figure 3:**
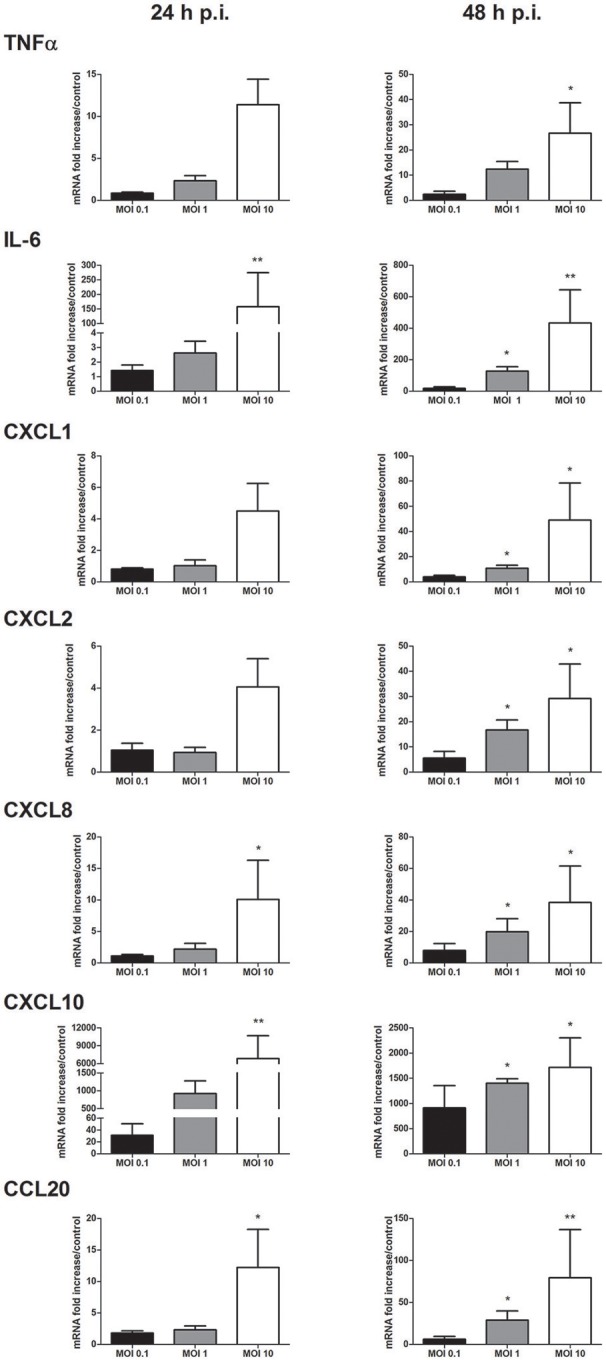
Cytokine and chemokine mRNA expression in response to keratinocyte infection by WNV. TNFα, IL-6, CXCL1, CXCL2, CXCL8, CXCL10, and CCL20 mRNA expression by human primary keratinocytes infected with WNV for 24 and 48 h, at MOI of 0.1, 1, and 10. mRNA expression levels are expressed as the fold increase above mock-infected cultures. Data are represented as mean + SEM of four independent experiments. ^*^p < 0.05 and ^**^p < 0.01.

The profile of inflammatory mediators induced by WNV infection was completed by focusing on cytokines and chemokines known to be expressed by keratinocytes. Messenger RNA expression of pro-inflammatory cytokines such as TNFα, IL-6 and chemokines such as CXCL1, CXCL2 CXCL8, CXCL10, and CCL20 was MOI- and time-dependently increased (Figure 3). Among them, CXCL10 was the most induced marker with mRNA levels 922 to 6,826-fold higher in WNV-infected cells compared to mock-infected cells at 24 h p.i.

### PRR expression in infected keratinocytes

**Figure 4:**
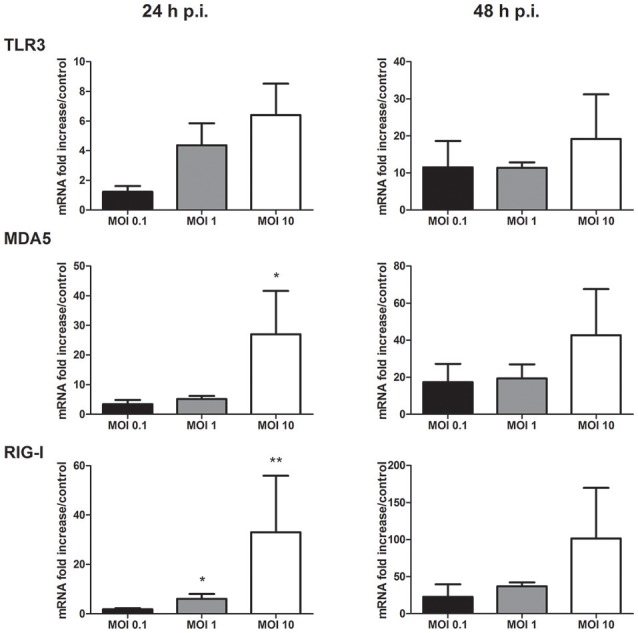
TLR3, MDA5, and RIG-I mRNA expression by human primary keratinocytes infected with WNV for 24 and 48 h, at MOI of 0.1, 1, and 10. mRNA expression levels are expressed as the fold increase above mock-infected cultures. Data are represented as mean + SEM of four independent experiments. ^*^p < 0.05 and ^**^p < 0.01.

As PRR signaling is crucial to initiate innate antiviral and proinflammatory responses following flaviviral infection, modulation of their expression during WNV replication was studied. The transmembranar TLR3, and the cytosolic helicases MDA5 and RIG-I PRR mRNA expression was increased in infected keratinocytes after 24 and 48 h of infection (Figure 4). PRR mRNA level increase was related to the infectious dose used, the stronger induction being observed at the MOI of 10 (Figure 4). TLR7 mRNA expression was not detected in infected as well as in mock-infected human primary keratinocytes (data not shown).

### Effect of *Ae. aegypti* saliva on WNV replication and the inflammatory response of infected keratinocytes

As WNV is a virus transmitted through the bite of a hematophagous arthropod, we wanted to assess the potential role of saliva from two distinct mosquito species on viral replication in human primary keratinocytes and on the inflammatory response induced. Saliva from *Ae. aegypti* or *Cx. quinquefasciatus* mosquitoes was thereby co-inoculated with WNV for keratinocyte infection, at a protein concentration of 0.5 μg/l that is consistent with physiological conditions of mosquito bites (Wasserman et al., 2004).

**Figure 5:**
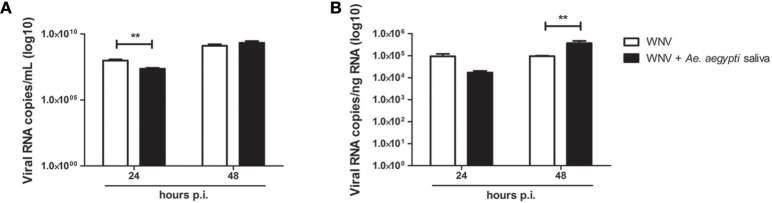
Effect of 0.5 μg/L of *Aedes aegypti* saliva on WNV replication. Viral loads were determined in cell supernatant (**A**, in log10 viral RNA copies/mL) and in cell lysates (**B**, in log10 viral RNA copies/ng RNA) at 24 and 48 h post-infection. Data are represented as mean + SEM of three independent experiments performed in duplicate. ^**^p < 0.01.

Keratinocyte infection with WNV in presence of *Ae. aegypti* saliva resulted in a decrease of WNV viral load assessed by RT-qPCR after 24 h of infection in cell supernatant as well as in cell lysate. Whereas at 48 h of infection, a significant increase of viral replication in cells infected in presence of saliva was noted in comparison to cells infected without saliva (Figure 5). A similar trend was observed with infective viral particle quantification of the supernatants by end-point dilution assay (Supplementary Figure 1).

**Figure 6:**
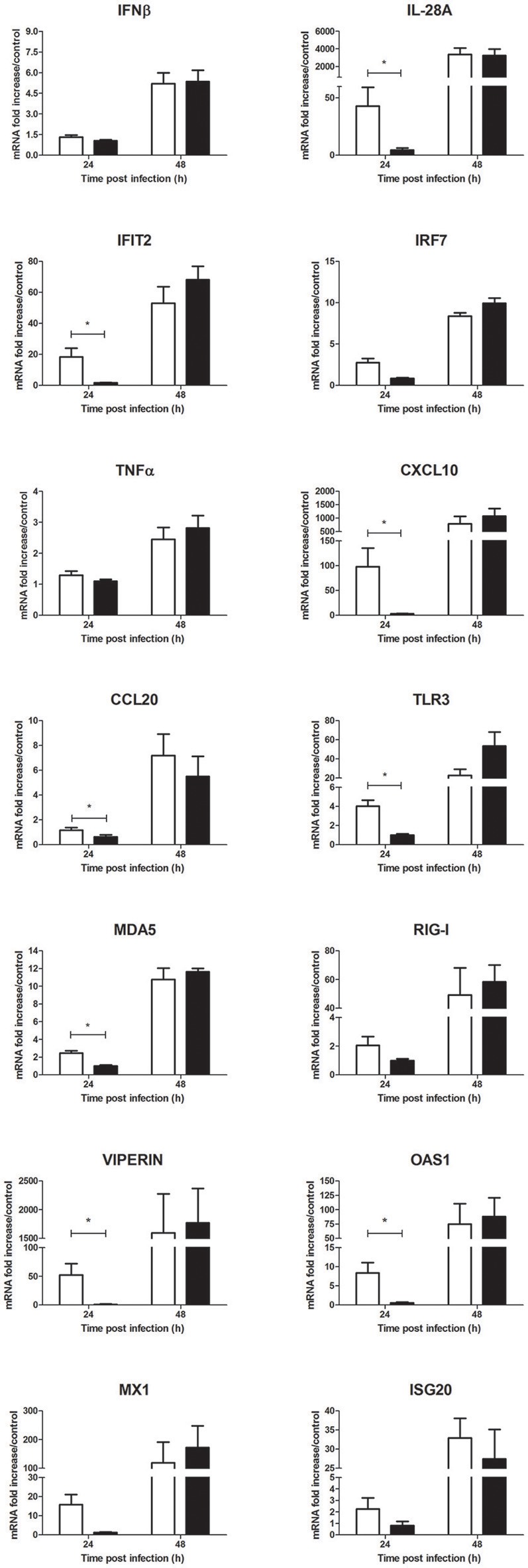
Effect of 0.5 μg/L of *Aedes aegypti* saliva on the WNV-induced inflammatory response during human primary keratinocyte infection. IFNβ, IL-28A, IFIT2, IRF7, TNFα, CXCL10, CCL20, TLR3, MDA5, RIG-I, viperin, OAS1, MX1, and ISG20 mRNA expression by keratinocytes infected with WNV at MOI of 1 for 24 and 48 h. Data are represented as mean + SEM of three independent experiments performed in duplicate. ^*^p < 0.05.

As a parallel to the viral replication, a significant decrease of inflammatory mediator mRNA levels such as IL-28A, IFIT2, CXCL10, and CCL20 was observed at 24 h p.i. in cells treated with *Ae. aegypti* saliva in comparison to cells infected without saliva (Figure 6). In presence of saliva, mRNA expression of macrophage colony-stimulating factor (M-CSF) was slighty reduced at 24 h and 48 h p.i. (Figure 6 and Supplementary Figure 2A). PRR mRNA expression tended also to decrease in presence of saliva at 24 h p.i. (Figure 6). This decrease also concerned more specific ISGs with previously described antiflaviviral activities such as viperin, 2′-5′-oligoadenylate synthetase 1 (OAS1), MX1 and ISG20 mRNA expression at early steps of infection (Figure 6). At the protein level, secretion of IFNβ was weakly reduced while that of CXCL10 was significantly inhibited in cells infected with *Ae. aegypti* saliva (Supplementary Figure 3).

Interestingly, *Ae. aegypti* saliva alone exerted an immunomodulatory effect on uninfected human primary keratinocytes after 24 h of stimulation by tending to inhibit the basal mRNA expression levels of MX1, OAS1, viperin, CCL20 and the PRRs (Supplementary Figure 4).

### Effect of *Cx. quinquefasciatus* saliva on WNV replication and the inflammatory response of infected keratinocytes

**Figure 7:**
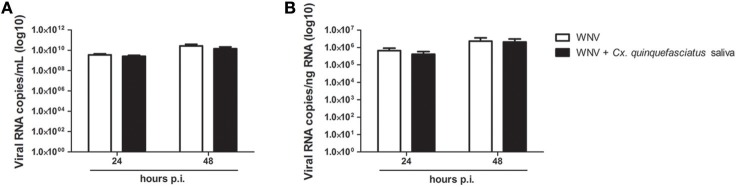
Effect of 0.5 μg/L of *Culex quinquefasciatus* saliva on WNV replication. Viral loads were determined in cell supernatant (**A**, in log10 viral RNA copies/mL) and in cell lysates (**B**, in log10 Viral RNA copies/ng RNA) from infected keratinocytes. Data are represented as mean + SEM of three independent experiments performed in duplicate.

Adjunction of *Cx. quinquefasciatus* saliva to the viral inoculum during keratinocyte infection did not significantly modulate viral load after 24 or 48 h of infection in cell supernatant or in cell lysate (Figure 7).

**Figure 8:**
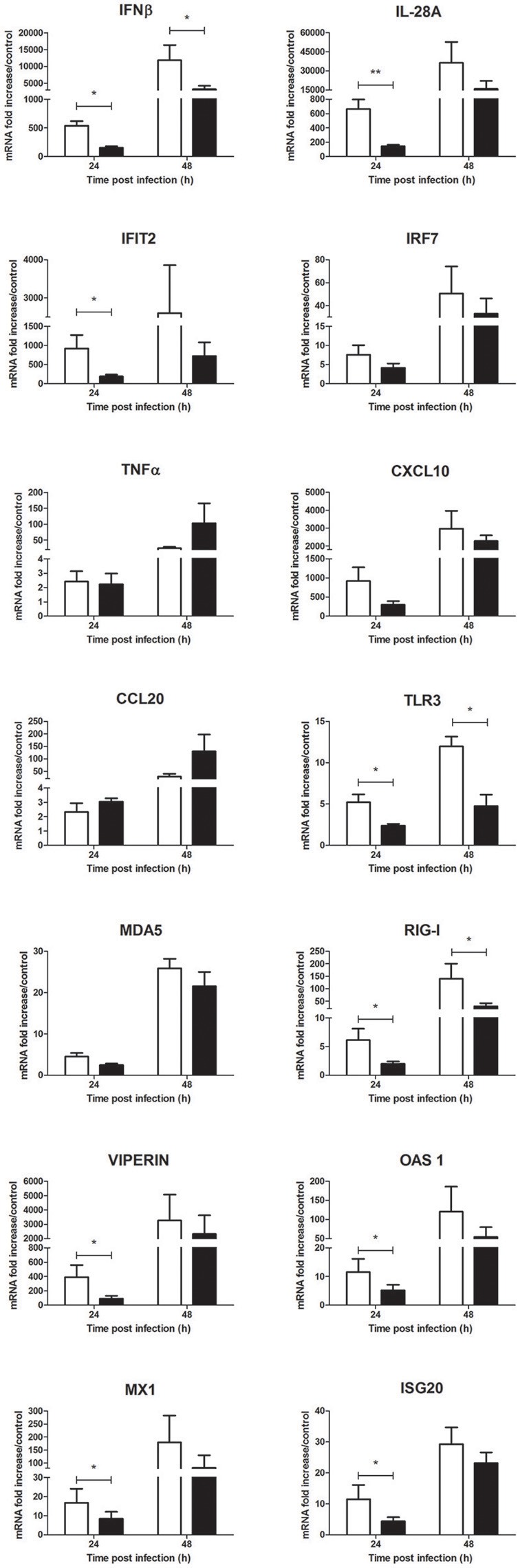
Effect of 0.5 μg/L of *Culex quinquefasciatus* saliva on the WNV-induced inflammatory response during human primary keratinocyte infection. IFNβ, IL-28A, IFIT2, IRF7, TNFα, CXCL10, CCL20, TLR3, MDA5, RIG-I, viperin, OAS1, MX1, and ISG20 mRNA expression by keratinocytes infected with WNV at MOI of 1 for 24 and 48 h. Data are represented as mean + SEM of three independent experiments performed in duplicate. ^*^p < 0.05 and ^**^p < 0.01.

Nonetheless, a significant decrease of inflammatory mediator expression such as IFNβ, IL-28A, IFIT2, M-CSF and the PRRs TLR3 and RIG-I was observed at 24 h p.i. and tend to be sustained at 48 h p.i. (Figure 8 and Supplementary Figure 2B). Messenger RNA levels of known antiflaviviral ISGs such as viperin, OAS1, MX1, and ISG20 also significantly decreased in presence of *Culex* saliva at 24 h p.i. (Figure 8). Finally, *Culex* saliva exert a significant inhibitory effect on the secreted levels of IFNβ and CXCL10 by WNV-infected keratinocytes (Supplementary Figure 5).

Contrary to *Ae. aegypti* saliva, *Cx. quinquefasciatus* saliva alone did not induce a decrease of ISG mRNA expression but tended to increase CCL20, IFNβ and IL-28A mRNA expression in uninfected human primary keratinocytes after 24 h of stimulation (Supplementary Figure 6).

## Discussion

WNV is transmitted through the inoculation by a mosquito bite mainly in the extravascular compartment of the dermis and the epidermis (Styer et al., 2007). Our study confirmed that primary human keratinocytes, the main cell type of the epidermis, were permissive to WNV as previously described by Lim et al, with an increase of viral load occurring mostly during the 48 first hours of infection (Lim et al., 2011). Dengue and Zika viruses, two other arboviruses belonging to the *Flavivirus* genus, were also shown to replicate in human keratinocytes so as in other skin cell types such as fibroblasts and dendritic cells (Hamel et al., 2015; Duangkhae et al., 2018). Unpublished data from our group demonstrate that WNV is also able to replicate in dermal fibroblasts and endothelial cells. Skin, thus, constitutes not only the first inoculation site but also the first replication site of WNV during infection. Moreover, in several cases, acute WNV infection is characterized only by dermatological manifestations, such as non-pruritic macular erythema, suggesting a tropism of the virus for the skin following viremic spread (Del Giudice et al., 2005).

The inflammatory response of human primary keratinocytes to WNV infection was described for the first time in this work. Keratinocytes are resident skin cells with innate immune functions, harboring numerous PRRs involved in the detection of PAMPs in order to initiate an inflammatory response to microbial infection (Briant et al., 2014). Hence, these epidermal cells are part of the first line of defense against WNV, as well as against other arboviruses, and act as sentinels thanks to their pathogen-sensing capacities and their privileged location in the largest interface of our body with the environment. A MOI-dependent inflammatory response was observed in keratinocytes at 24 h p.i. mainly involving pleiotropic cytokines, such as TNFα and IL-6, various chemokines, type I and III IFNs, and ISGs such as IFIT proteins. IFNs and IFN-dependent mediators are known to be strongly induced during viral infection and exert potent antiviral activities (Lazear et al., 2011; Lazear and Diamond, 2015). Type I IFN is thought to control WNV infection by (i) the induction of cell-intrinsic antiviral effectors, known as ISGs, restricting different steps of virus replication and (ii) by activating the adaptive immune (Lazear et al., 2011; Lazear and Diamond, 2015). It has been shown that cell IFN-treatment before infection or addition of IFN to WNV already infected cells, reduced viral replication (Isaacs and Westwood, 1959; Samuel and Diamond, 2005) and that in mice lacking type I IFN receptor, WNV infection spread faster than in wild type mice leading to 100% of mortality (Samuel and Diamond, 2005). Type III IFNs are also members of IFN family with antiflaviviral properties (Lazear et al., 2015; Douam et al., 2017). However, IFNλ exerts a weaker antiviral effect against WNV than type I IFN in two human cell lines (Ma et al., 2009). In our work, type I and III IFN expression was induced in human primary keratinocytes according to MOI and time of infection suggesting that they could play a role against WNV during cutaneous infection. Moreover, type I and III IFNs induce and regulate expression of ISGs. Induction of several ISG mRNA expression such as IFIT-1 to 3, viperin, OAS1, MX1 and ISG20, was observed in WNV-infected keratinocytes. It has been suggested that viperin and ISG20 inhibited steps in viral proteins and/or viral RNA biosynthesis during human embryonic kidney 293 cell infection with WNV (Jiang et al., 2010; Szretter et al., 2011), whereas OAS1 polymorphism was associated with WNV infection susceptibility in mice and humans (Mashimo et al., 2002; Kajaste-Rudnitski et al., 2006; Lim et al., 2009; Szretter et al., 2011). As regards IFIT proteins, they own a broad-spectrum antiviral activity helping to control viral replication and pathogenesis. They have been demonstrated to regulate protein translation through several mechanisms such as by interacting with eIF3 (Hui et al., 2005; Terenzi et al., 2006), binding of uncapped or incompletely capped viral RNA, and sequestering viral RNA or proteins in the cytoplasm (Diamond and Farzan, 2013). They might also interplay in regulation of intrinsic and extrinsic cell immune responses (Diamond and Farzan, 2013). *In vivo*, a tissue-specific antiviral effect of IFIT2 has previously been described in a mouse model of central nervous system WNV infection (Cho et al., 2013). Nonetheless, flaviviruses such as WNV have developed strategies to subvert IFIT1 and IFIT2 functions using 2′-O methyltransferase activity (Daffis et al., 2010; Szretter et al., 2011). In conclusion, although human keratinocytes are permissive to WNV, they express specific antiviral proteins during infection to limit viral replication in the skin.

The innate antiviral cellular response involves IRF-family transcription factors as regulators of host defense by inducing production of IFNs and ISGs. In human keratinocytes, Kalali *et al*. reported a constitutive expression of IRF3 whereas expression of IRF1, IRF2, and IRF7 was inducible (Kalali et al., 2008). IRF3 and IRF7 have been described involved in protection against WNV infection in mouse models (Daffis et al., 2007, 2008). Our results showed that WNV infection of primary keratinocytes induced an increase of IRF7 expression without modulating IRF3 mRNA levels. This has been previously reported in human skin cells infected with the other flaviviruses, DENV or ZIKV (Surasombatpattana et al., 2011; Hamel et al., 2015). These results could suggest a major role of IRF7 in host skin immune response even if we cannot exclude a role of IRF3 as its expression is constitutive.

IRF3 and IRF7 are phosphorylated after recruitment of the adaptor molecule interferon promoter-stimulating factor 1 (IPS-1) by dsRNA-activated RLRs, MDA5 and RIG-I (Sato et al., 2000; Kato et al., 2006). Keratinocyte infection with WNV was also associated with an increase of TLR3, RIG-I and MDA5 mRNA expression, this phenomenon may contribute to an amplification loop facilitating antiviral ISG expression. If the role of TLR3 in WNV sensing has been controversial (Fredericksen et al., 2004, 2008; Wang et al., 2004; Fredericksen and Gale, 2006; Daffis et al., 2008), MDA5 and RIG-I have been described cooperating in WNV sensing in a time-dependent manner, involving first RIG-I and then MDA5 (Fredericksen et al., 2004, 2008; Fredericksen and Gale, 2006). Moreover, the role of TLR7 in WNV sensing has been reported *in vitro*, considering a higher viral replication and weaker cytokine expression in keratinocytes from TLR7^−/−^ mice than in those from wild-type mice (Welte et al., 2009). TLR7 response following cutaneous WNV infection has also been suggested to promote Langerhans cell migration from the skin to the draining lymph nodes (Welte et al., 2009). Nonetheless,*in vivo*, no difference has been observed regarding susceptibility to WNV encephalitis and in blood or brain RNA viral loads, between wild-type and TLR7^−/−^ mice (Welte et al., 2009). In unstimulated primary human keratinocytes, TLR7 has been reported to be unexpressed but its expression was induced following stimulation with synthetic viral ds RNA analog poly(I:C) (Kalali et al., 2008). In our primary cultures, TLR7 was neither constitutively expressed, nor induced following WNV infection, suggesting that this receptor is not involved in WNV sensing by keratinocytes from the basal layer of the epidermis. However, we cannot exclude a role of TLR7 during WNV infection of the whole epidermis as TLR7 has been described as being constitutively expressed in the human epidermal layers with the exception of the basal layer in normal skin biopsies (Donetti et al., 2017).

The inflammatory response induced in infected human primary keratinocytes also consisted in mRNA level increase of the pleiotropic cytokines TNFα and IL-6 as well as chemokines, such as CXCL1, CXCL2, CXCL8, CXCL10, and CCL20. CCL20 plays an important role in the homing of lymphocytes and dendritic cells to inflammation site, and CXCL1, CXCL2, and CXCL8 are polymorphonuclear (PMN) leucocytes attracting chemokines. CXCL1 mRNA expression has been described to be induced by WNV infection *in vitro* (Quick et al., 2014) and *in vivo* (Kumar et al., 2014). PMN leucocytes have been suggested to play a biphasic role, deleterious first and then beneficial, in pathophysiology of WNV infection (Bai et al., 2010). CXCL10 is induced in several models of WNV brain infection as well as in WNV-infected blood donors (Cheeran et al., 2005; Klein et al., 2005; Garcia-Tapia et al., 2007; Tobler et al., 2008; Qian et al., 2011; Quick et al., 2014; Bielefeldt-Ohmann et al., 2017). This T-cell chemoattractant chemokine was the most strongly induced mediator during keratinocyte infection and it was reported that the plasmatic level of this protein was higher among WNV-infected individuals with a better outcome (Hoffman et al., 2016). CXCL10 as well as TNFα are involved in CD8 (+) T cell traffic playing a major role in WNV clearance (Klein et al., 2005; Shrestha et al., 2008) and protecting against WNV spread. TNFα also possesses widespread antiviral effects (Benedict, 2003).

Taken together, our results highlight the role of primary keratinocytes as innate immune cells able to establish a cutaneous antiviral response and attract different types of leukocytes, including PMN, T and dendritic cells, in the area of virus inoculation.

WNV contained in a mosquito's salivary glands is inoculated with saliva during mosquito blood meal after probing the skin (Styer et al., 2007; Choumet et al., 2012). Because of mosquito saliva wide properties and as skin is the first site of WNV replication, we assessed the potential role of mosquito saliva on WNV replication and inflammatory response induced in infected human primary keratinocytes.

On the one hand, adjunction of *Ae. aegypti* saliva resulted in a two-step phenomenon regarding WNV replication, a first inhibitory effect at an early time of infection and, later on, a proviral one. It is known that some identified *Ae. aegypti* saliva proteins such as aegyptin or D7 proteins can interfere with DENV replication (McCracken et al., 2014; Conway et al., 2016). Previously, during DENV and CHIKV infection of human primary keratinocytes and human fibroblasts, respectively, in presence of *Ae. aegypti* saliva product, a proviral effect was also reported (Surasombatpattana et al., 2014; Wichit et al., 2017).

On the other hand, adjunction of *Cx. quinquefasciatus* saliva did not have any consequence on WNV replication in keratinocytes. Styer et al. have demonstrated that *in vivo*, mosquito-infected chickens had higher WNV viremia and viral shedding as compared to those infected with needles in the absence of saliva (Styer et al., 2006). In a mouse model, animals previously exposed to uninfected-mosquito bites before being infected with WNV exhibited higher viremia, tissue-titers and neurological symptoms than those infected in the absence of mosquito saliva suggesting that *Culex* saliva could also exert a proviral effect in mammals (Styer et al., 2011). Nonetheless, at the inoculation site, there was no increase of viral titer in skin of mosquito-vs. needle-infected mice at any time of infection and even a decrease of viral titer at 24 h of infection (Styer et al., 2011) suggesting that *Culex* saliva is also not proviral in mice's skin. Congruently, our results suggest that human keratinocytes are not sensitive to the proviral effect of *Culex* saliva.

Concerning the effect of saliva on uninfected human primary keratinocytes, *Ae. aegypti* saliva tended to reduce the basal levels of antiviral ISGs at 24 and 48 h post-stimulation. Interestingly, this anti-inflammatory effect was not found using *Cx. quinquefasciatus* saliva. These results are consistent with a previous work of Wanasen *et al*. demonstrating that salivary gland extract (SGE) from *Ae. aegypti* induced a significant decrease of cell proliferation and cytokine production in murine splenocytes contrary to *Cx. quinquefasciatus* SGE (Wanasen et al., 2004). It has been suggested that according to the concentration of SGE delivered at the site of injection, the immunomodulatory effect observed could change, thereby creating a local and differential immunological environment according to the distance from the site of the bite (Wasserman et al., 2004; Styer et al., 2011). The differential immunomodulatory effect of *Culex* and *Aedes* saliva has been suggested as being related to vector's host preferences, as *Culex* are preferentially ornithophilic mosquitoes whereas *Aedes* more anthropophilic species (Wanasen et al., 2004; Schneider and Higgs, 2008). Therefore, we hypothesized that the immunomodulatory effect of *Culex* saliva would probably be more specific to bird species. Furthermore, *Aedes* and *Culex* mosquito saliva do not exhibit the same salivary protein composition and biological effects (Ribeiro, 2000). During WNV infection of human primary keratinocytes, the immunomodulatory effect of *Aedes* and *Culex* saliva was observed mainly at 24 h p.i. at the transcriptomic level and also at 48 h p.i. for IFNβ and CXCL10 secretion. Schneider & Higgs have hypothesized that mosquito saliva could impair immune response to arbovirus through downregulation of Th1 and antiviral cytokines (Schneider and Higgs, 2008). A few studies have assessed the effect of *Ae. aegypti* saliva on skin cell inflammatory response in the context of flaviviral infections. Decreased expression of type I IFN-responsive genes was observed in presence of *Ae. aegypti* saliva (Surasombatpattana et al., 2014; Wichit et al., 2017). In this study, decreased expression of IFNs and known antiflaviviral ISGs, including MX1, OAS1, ISG20 and viperin was observed in presence of both *Aedes* and *Culex* saliva after 24 h of keratinocyte infection. Inhibition of the keratinocyte antiviral response by mosquito saliva could favor the viral replication which may involve other skin cells surrounding keratinocytes such as Langerhans cells, melanocytes or deeper dermal mast cells and fibroblasts. Finally, a decrease of M-CSF mRNA, a cytokine involved in macrophage differentiation and proliferation, was noted during keratinocyte infection in presence of saliva. As macrophages are known to play a protective role against WNV infection (Ben-Nathan et al., 1996; Bryan et al., 2018), the inhibitory effect of mosquito saliva on M-CSF expression could also result in a lessening of the immune response favorable to virus propagation.

## Conclusion

This work highlights for the first time the inflammatory response of human primary keratinocytes infected by WNV. These skin resident cells permissive to WNV, are able to sense viral PAMPs and initiate immune response through expression of various inflammatory mediators and antiviral effectors. This may lead to prompt induction of an antiviral state in infected as well as in uninfected neighboring cells, thereby helping to limit viral spread. We also showed that the saliva from two potential vectors of the infection exerts an inhibitory effect on the antiviral response of WNV-infected keratinocytes with adverse effects enhancing knowledge of WNV pathophysiology in the skin.

## Author contributions

MG, NL, and CB conceived and designed the study. MG performed the experiments and wrote the draft. HA and MW provided *Culex* saliva. FD, MB, and DM provided *Aedes* saliva. AD performed some transcriptomic analysis. CB and NL contributed to the revision of the manuscript. All authors approved the final version of the manuscript.

### Conflict of interest statement

The authors declare that the research was conducted in the absence of any commercial or financial relationships that could be construed as a potential conflict of interest.
