# Supplementary material for: Innate Immune Response of Primary Human Keratinocytes to West Nile Virus Infection and Its Modulation by Mosquito Saliva
Source: Front Cell Infect Microbiol. 2018 Nov 2;8:387. doi: 10.3389/fcimb.2018.00387 (PMC6224356; doi:10.3389/fcimb.2018.00387)
Supplement: Supplementary file 1 [file Table_1.docx]

Supplementary Material

**Innate immune response of primary human keratinocytes to West Nile virus infection and its modulation by mosquito saliva**

**Magali GARCIA^1,2^, Haoues ALOUT^3^, Fodé DIOP^4^, Alexia DAMOUR^2^, Michèle BENGUE^4^, Mylène WEILL^3^, Dorothée MISSE^4^, Nicolas LEVEQUE^1,2^ and Charles BODET^2^**

**Correspondence:** Charles Bodet, [charles.bodet@univ-poitiers.fr](mailto:charles.bodet@univ-poitiers.fr)


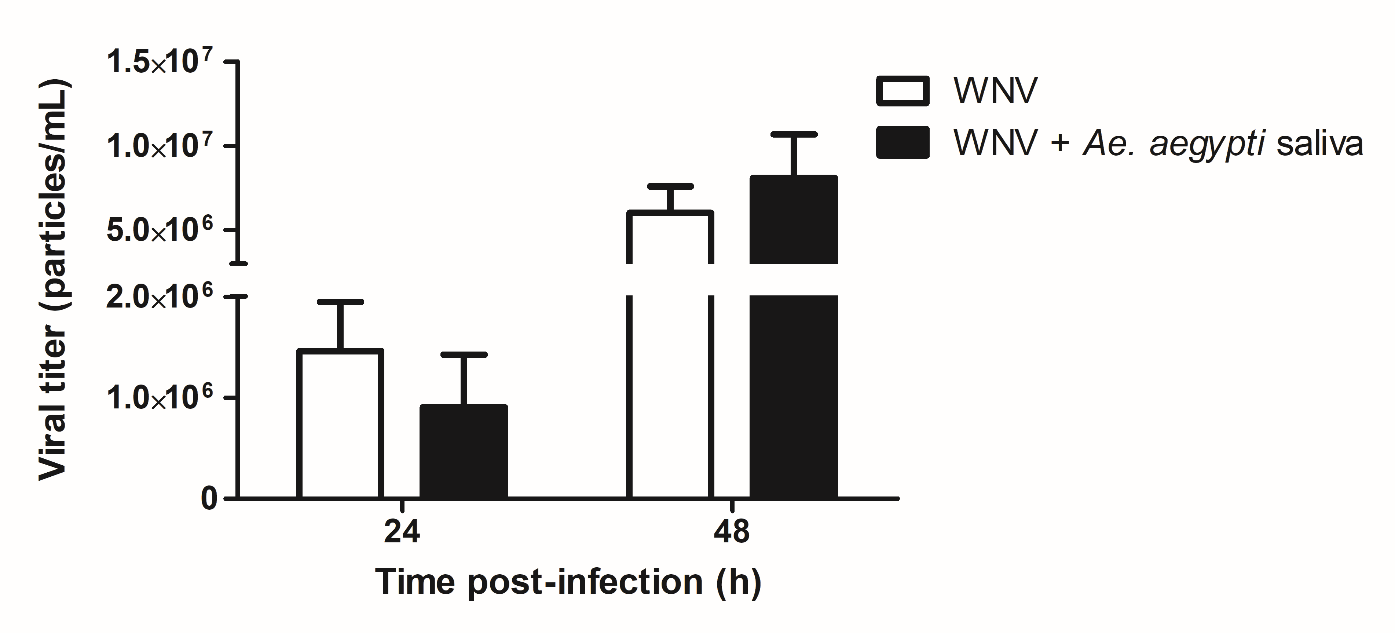


**Supplementary Figure 1.** Effect of 0.5 µg/L of *Aedes aegypti* saliva on WNV replication. Viral titers were determined in cell supernatant by end-point dilution assay at 24 h and 48 h post-infection. Data are represented as mean + SEM of three independent experiments performed in duplicate.


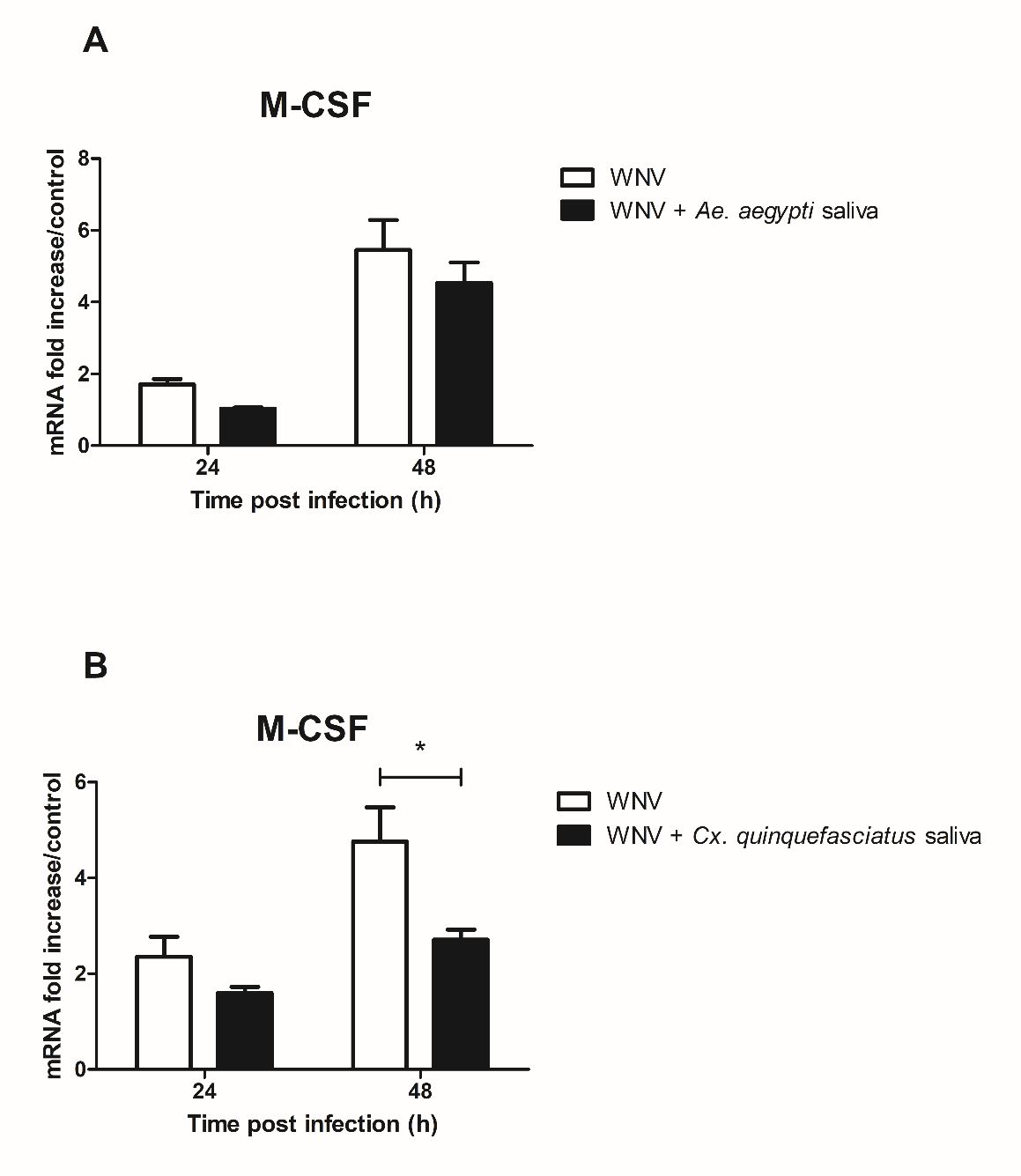


**Supplementary Figure 2.** Effect of 0.5 µg/L of *Aedes aegypti* (**A**) or *Culex quinquefasciatus* (**B**) saliva on the WNV-induced inflammatory response during human primary keratinocyte infection. M-CSF mRNA expression by keratinocytes infected with WNV at MOI of 1 for 24 h and 48 h. Data are represented as mean + SEM of three independent experiments performed in duplicate. *p< 0.05.


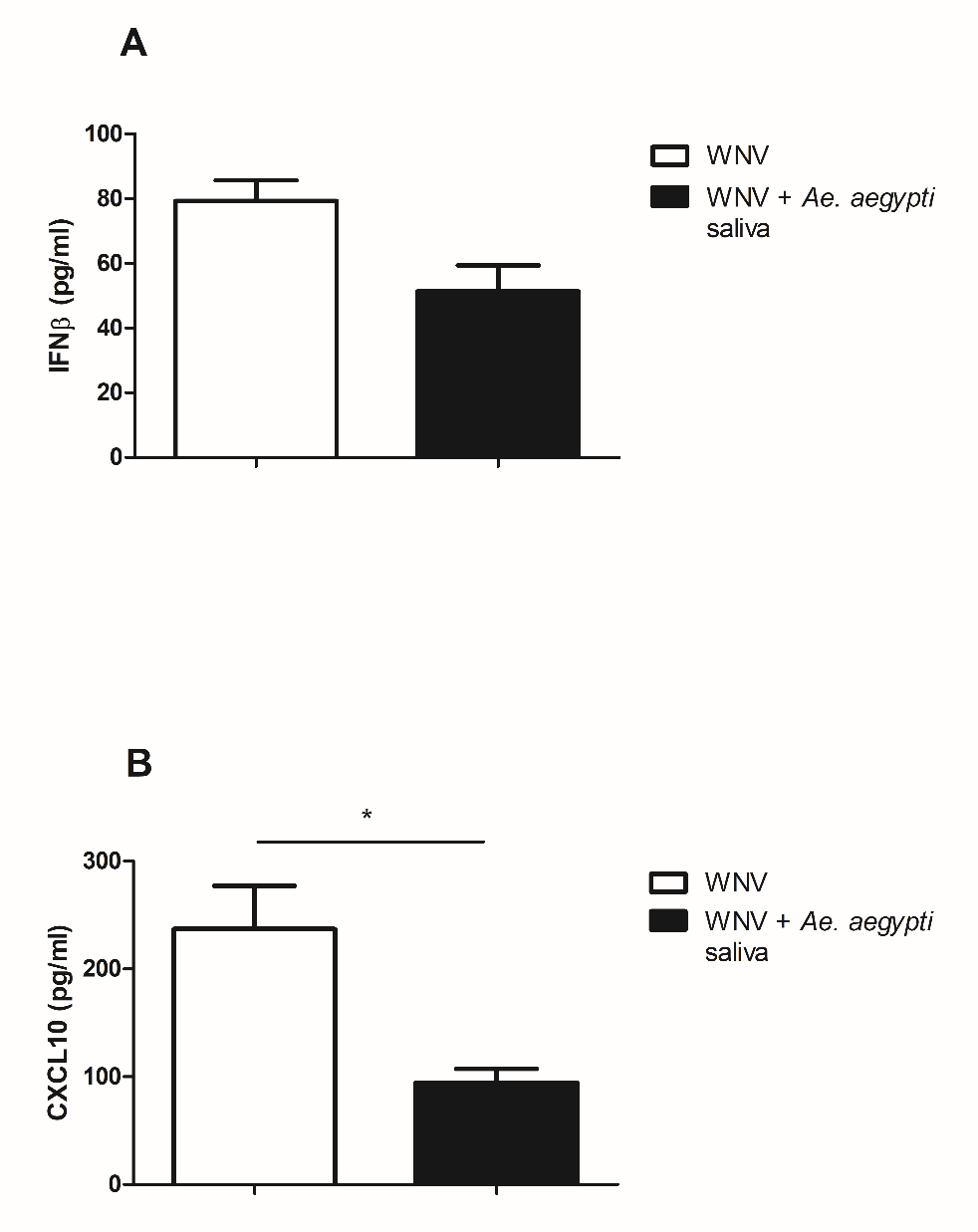


**Supplementary Figure 3.** Effect of 0.5 µg/L of *Aedes aegypti* saliva on the WNV-induced IFNβ and CXCL10 secretion during human primary keratinocyte infection. Protein concentrations (pg/mL) were measured by ELISA assays in culture supernatants of keratinocytes infected with WNV at MOI of 1 for 48 h. Data are represented as mean + SEM of three independent experiments performed in duplicate. *p< 0.05.


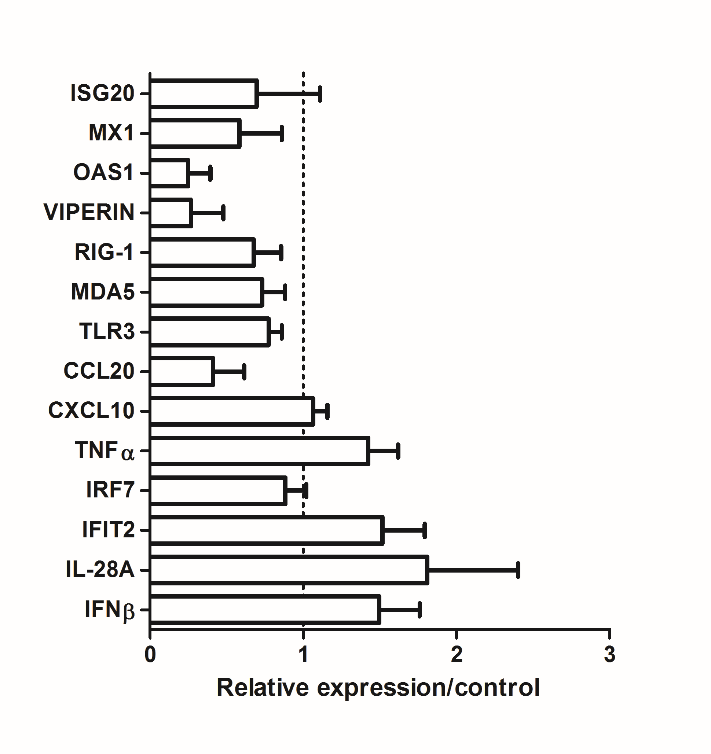


**Supplementary Figure 4.** Effect of 0.5 µg/L of *Aedes aegypti* saliva on uninfected human primary keratinocytes after 24 h of stimulation. mRNA expression levels are expressed as the fold increase above unstimulated cultures. Data are represented as mean + SEM of three independent experiments realized in duplicate.


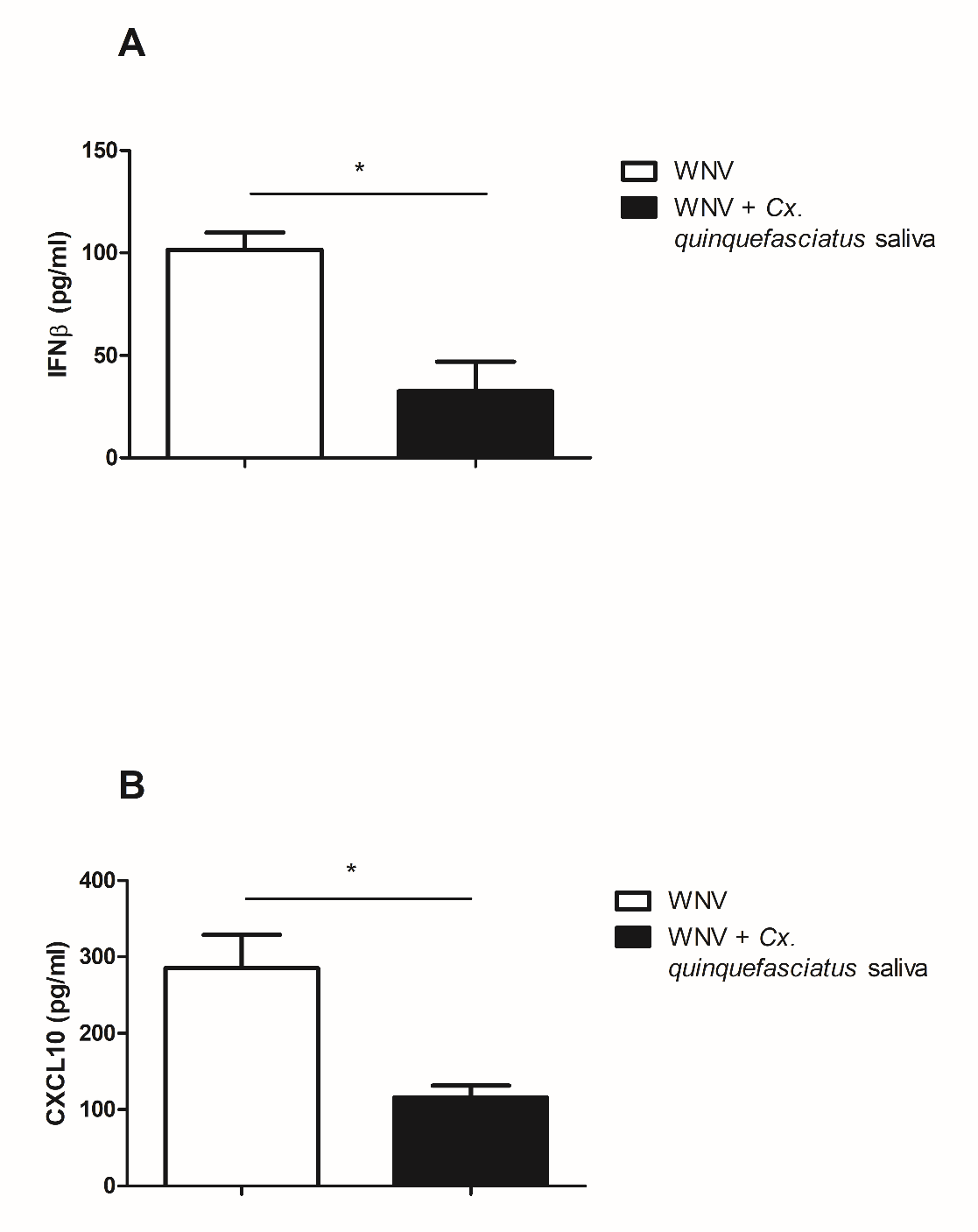


**Supplementary Figure 5.** Effect of 0.5 µg/L of *Culex quinquefasciatus* saliva on the WNV-induced IFNβ and CXCL10 secretion during human primary keratinocyte infection. Protein concentrations (pg/mL) were measured by ELISA assays in culture supernatants of keratinocytes infected with WNV at MOI of 1 for 48 h. Data are represented as mean + SEM of three independent experiments performed in duplicate. *p< 0.05.

**
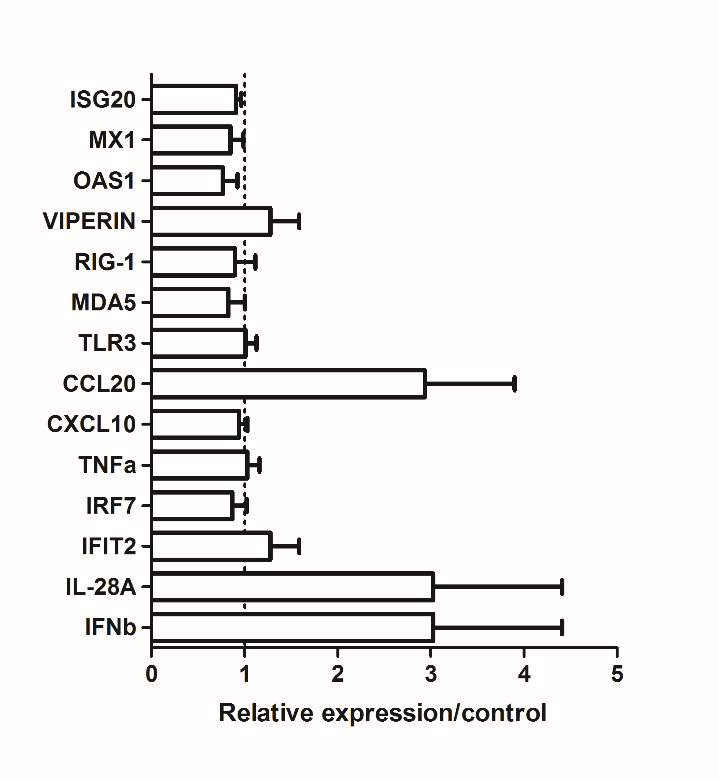
**

**Supplementary Figure 6.** Effect of 0.5 µg/L of *Culex quinquefasciatus* saliva on uninfected human primary keratinocytes after 24 h of stimulation. mRNA expression levels are expressed as the fold increase above unstimulated cultures. Data are represented as mean + SEM of three independent experiments realized in duplicate.
